# Molecular Characterisation and Functions of Fis1 and PDCD6 Genes from *Echinococcus granulosus*

**DOI:** 10.3390/ijms19092669

**Published:** 2018-09-08

**Authors:** Ning Wang, Jiafei Zhan, Cheng Guo, Chunyan Li, Nengxing Shen, Xiaobin Gu, Yue Xie, Xuerong Peng, Guangyou Yang

**Affiliations:** 1 Department of Parasitology, College of Veterinary Medicine, Sichuan Agricultural University, Chengdu 611130, China; wangningzhuhui@hotmail.com (N.W.); zhanjiafei@hotmail.com (J.Z.); chengguo_92@hotmail.com (C.G.); chunyanli1223@163.com (C.L.); nengxing1991@hotmail.com (N.S.); guxiaobin198225@126.com (X.G.); zhandegaokandey123@163.com (Y.X.); 2 College of Bioengineering, Sichuan University of Science and Engineering, Yibin 644000, China; 3 Department of Chemistry, College of Life and Basic Science, Sichuan Agricultural University, Chengdu 611130, China; gujiang1205@yeah.net

**Keywords:** mitochondrial fission protein 1, programmed cell death protein 6, *Echinococcus granulosus*, infertile cyst, apoptosis, oxidative stress

## Abstract

Cystic echinococcosis, a parasitic zoonosis that causes significant economic losses and poses a threat to public health, is caused by larvae of the tapeworm *Echinococcus granulosus*. Infection causes infertile cysts in intermediate hosts that cannot produce protoscoleces (PSCs) or complete the life cycle. Herein, we cloned, expressed, and characterised mitochondrial fission protein 1 (*Eg-Fis1*) and programmed cell death protein 6 (*Eg-PDCD6*) from *E. granulosus*, and explored their functions related to infertile cysts. *Eg-Fis1* and *Eg-PDCD6* encode putative 157 and 174 residue proteins, respectively, and Western blotting indicated good reactogenicity for both. Eg-Fis1 and Eg-PDCD6 were ubiquitously distributed in all stages of *E. granulosus*. Furthermore, mRNAs of Eg-Fis1 and Eg-PDCD6 were upregulated following H_2_O_2_ treatment which induced apoptosis in PSCs. To investigate the regulation of apoptosis in response to oxidative stress, RNA interference (RNAi) and terminal deoxynucleotidyl transferase dUTP nick-end labelling (TUNEL) assays were performed. The apoptotic rate of the Eg-Fis1 RNAi group was significantly lower than non-interference group, but there was no such difference for Eg-PDCD6. In conclusion, Eg-Fis1 promotes apoptosis induced by oxidative stress, whereas Eg-PDCD6 does not appear to be a key regulator of apoptosis.

## 1. Introduction

The larval stage of *Echinococcus granulosus* (Platyhelminthes, Cestoda) causes cystic echinococcosis (CE), which is a significant global public health concern in many rural, grazing areas of South America, North and East Africa, and Australia [1,2]. This parasitic disease is listed as one of the neglected tropical diseases in the World Health Organisation (WHO) “Accelerating work to overcome the global impact of neglected tropical diseases—a roadmap for implementation” document [3]. A wide range of domestic and wild mammals, as well as humans, can serve as intermediate hosts in the life cycle of this parasite [1]. Hydatid cysts develop in the intermediate host, mainly in internal organs, such as liver and lungs, and cause compression, mechanical damage, or even death [3,4]. Two types of hydatid cysts occur; fertile cysts, in which protoscoleces (PSCs) are found both joined to the germinal layer and free in the hydatid fluid filling the cyst cavity, and infertile cysts, which do not produce PSCs and are, therefore, unable to complete the life cycle of the parasite [5,6,7].

Mitochondrial fission protein 1 (Fis1) is known to mediate mitochondrial fission, which plays a key role in the process of mitochondrial membrane disruption [8,9,10]. Mitochondrial fusion and fission processes are vital for maintenance of organellar morphology and distribution, but also for cell proliferation and cell differentiation [10]. A previous study showed that increasing mitochondrial fission can promote apoptosis. Programmed cell death protein 6 (PDCD6), also known as apoptosis-linked gene-2 (ALG-2), is a calcium-binding modulator protein involved in cell proliferation and death [11,12]. It was identified in a cell death trap assay as a pro-apoptotic protein in a functional screen of T-cell hybridoma cells, and it was linked to calcium-dependent apoptosis [13,14,15,16]. Fis1 and PDCD6 have been reported to play an important role in promoting apoptosis in organisms [17,18]. However, their functions in *E. granulosus* are not yet clear.

In the present study, genes encoding Fis1 and PDCD6 from *E. granulosus* were cloned, expressed, and characterised. Their locations during different life cycle stages were investigated by immunolocalisation, gene expression following H_2_O_2_ treatment was evaluated, and their functions in PSCs, in vitro, were explored using RNA interference (RNAi).

## 2. Results

### 2.1. Bioinformatic Analysis of Eg-Fis1 and Eg-PDCD6

The full-length *Eg-Fis1* cDNA comprises a 474 bp gene encoding a protein of 157 amino acids (aa) with no signal peptide, but with a single transmembrane region (aa 129−157). The predicted molecular weight (MW) of *Eg-Fis1* is 16.93 kDa. The cDNA sequence of *Eg-PDCD6* contains an open reading frame (ORF) of 525 bp encoding a polypeptide of 174 aa without a signal peptide or a transmembrane region, and the deduced protein has a MW of 20.34 kDa. InterPro analysis showed that *Eg-Fis1* has two functional domains comprising a tetratricopeptide-like helical domain (aa 26−146) and a C-terminal tetratricopeptide repeat domain (aa 78−120), while *Eg-PDCD6* has an EF-hand domain (aa 7−169).

**Figure 1:**
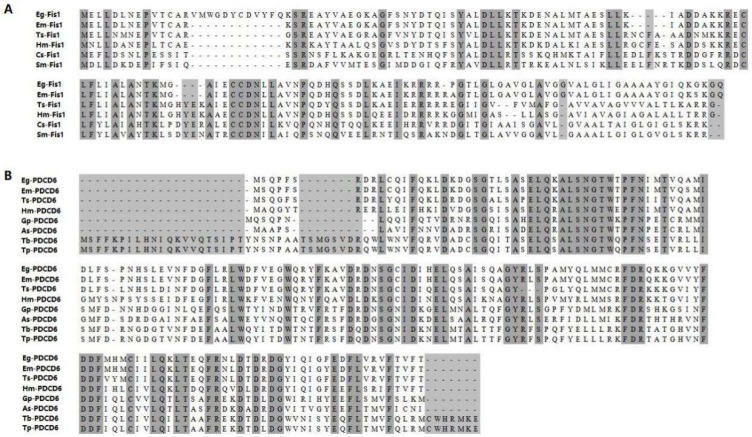
Sequence alignments of *Eg-Fis1* and *Eg-PDCD6* with homologs from other species. (**A**) Accession numbers for Fis1 are as follows: *Echinococcus granulosus* (GeneDB: EgrG 000225700); *Echinococcus multilocularis* (GeneDB: EmuJ 000225700); *Taenia solium* (GeneDB: TsM 000708100); *Hymenolepis microstoma* (GeneDB: HmN 000627400); *Clonorchis sinensis* (NCBI: GAA51930); *Schistosoma mansoni* (GeneDB: Smp 032230); (**B**) Accession numbers for PDCD6 are as follows: *Echinococcus granulosus* (GeneDB: EgrG_000715700); *Echinococcus multilocularis* (GeneDB: EmuJ_000715700); *Taenia solium* (GeneDB: TsM_000243100); *Hymenolepis microstoma* (GeneDB: HmN_000969200); *Globodera pallida* (GeneDB: GPLIN_000021400); *Ascaris suum* (NCBI: ADY44670.1); *Trichinella britovi* (NCBI: KRY46020.1); *Trichinella patagoniensis* (NCBI: KRY14254.1).

Amino acid sequences of *Fis1* and *PDCD6* from various species were retrieved from GenBank and GeneDB database, and multiple sequence alignment revealed that *Fis1* genes are highly variable. *Eg-Fis1* shares highest sequence identity with *Echinococcus multilocularis Fis1* (90.51%), followed by 63.03% with *Hymenolepis microstoma Fis1*, and 38.32% and 34.13% with *Clonorchis sinensis* and *Schistosoma mansoni*, respectively (Figure 1). By contrast, *PDCD6* genes among species are highly conserved. *Eg-PDCD6* is most similar to *E. multilocularis PDCD6* (98.85%), and shares 89.08% and 71.43% sequence identity with *PDCD6* from *Taenia solium* and *H. microstoma*, respectively, but shares lower identity with sequences from other nematodes, such as *Globodera pallida* and *Ascaris suum* (Figure 1).

**Figure 2:**
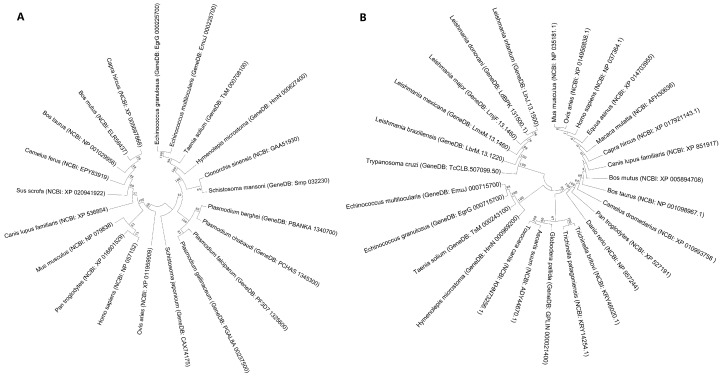
Phylogenetic (neighbour-joining) trees of Eg-Fis1 and Eg-PDCD6. (**A**) Phylogenetic analysis of *Eg-Fis1* and its homologs; (**B**) Phylogenetic analysis of *Eg-PDCD6* with and its homologs.

Evolutionary analysis of Fis1 aa sequences showed that *Eg-Fis1* shares relatively high homology with *Fis1* from congeneric Cestodes and Nematoda, except for *Schistosoma japonicum.* Plasmodium formed an independent branch close to Cestoda and Trematoda, while mammals formed another branch (Figure 2A). A phylogenetic neighbour-joining (NJ) tree was constructed for *PDCD6* genes based on *PDCD6* aa sequences obtained from databases. The phylogenetic tree of sequences from 27 species forms four main branches (Cestoda, Nematoda, protozoan, and mammals), with *Eg-PDCD6* in the Cestoda branch (Figure 2B).

### 2.2. Expression of Recombinant rEg-Fis1 and rEg-PDCD

**Figure 3:**
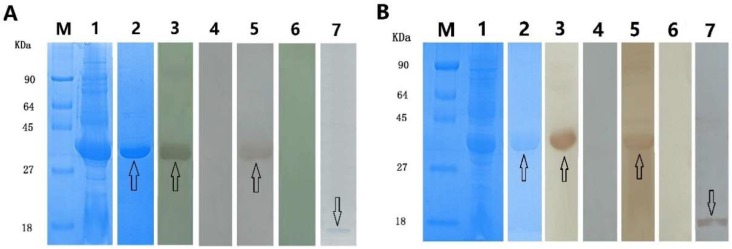
SDS-PAGE and Western blotting analysis of rEg-Fis1 and rEg-PDCD6. (**A**) Immune recognition of rEG-Fis1; (**B**) Immune recognition of rEG-PDCD6. M, molecular mass markers (kDa); lane 1, total proteins from *Escherichia coli* BL21 (DE3) transformants harbouring pET32a(+)-Eg-Fis1/pET32a(+)-Eg-PDCD6 induced by isopropyl-β-d-1-thiogalactopyranoside (IPTG); lane 2, purified recombinant protein; lane 3, purified rEg-Fis1/rEg-PDCD6 probed with anti-rEg-Fis1/anti-rEg-PDCD6 rabbit sera; lane 4, purified rEg-Fis1/rEg-PDCD6 probed with pre-immunised rabbit sera; lane 5, purified rEg-Fis1/rEg-PDCD6 probed with sera from cystic echinococcosis (CE)-positive sheep sera; lane 6, purified rEg-Fis1/rEg-PDCD6 probed with non-infected sheep sera; lane 7, total protoscoleces (PSCs) extract probed with anti-Eg-Fis1/anti-Eg-PDCD6 rabbit sera IgG.

The *Eg-Fis1* and *Eg-PDCD* genes were amplified from PSCs, and recombinant rEg-Fis1 and rEg-PDCD proteins were successfully expressed in *Escherichia coli*. After purification, the His-tagged rEg-Fis1 protein yielded a single band close to the predicted size of 35 kDa following separation by 15% sodium dodecyl sulphate-polyacrylamide gel electrophoresis (SDS-PAGE), while the rEg-PDCD6 band was close to the expected size of 37 kDa (Figure 3).

In Western blotting assays, both rEg-Fis1 and rEg-PDCD reacted with cystic echinococcosis (CE)-positive sheep sera, and with anti-Eg-Fis1 and anti-Eg-PDCD6 rabbit sera, respectively (Figure 3). Moreover, anti-Eg-Fis1 and anti-Eg-PDCD6 rabbit sera IgGs recognised their respective native proteins in PSC extracts. As expected, specific bands were not observed following incubation with sera from non-infected sheep or pre-immunised rabbit.

### 2.3. Localisation of Eg-Fis1 and Eg-PDCD6 during Different E. granulosus Life Cycle Stages

**Figure 4:**
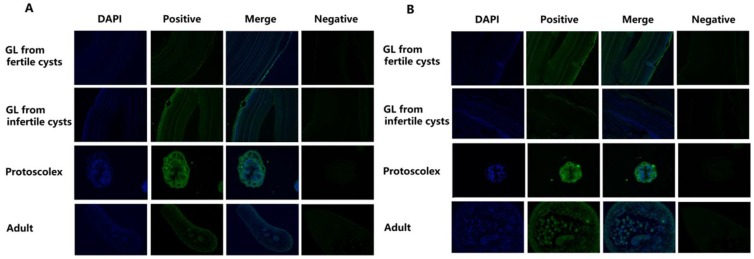
Immunohistochemical localisation of Eg-Fis1 and Eg-PDCD6 in different life cycle stages of *E. granulosus*. (**A**) Immunohistochemical localisation of Eg-Fis1; (**B**) Immunohistochemical localisation of Eg-PDCD6. Images of PSCs are magnified at 400×, and cyst walls and adult worms are magnified at 200×. The nucleus was stained with DAPI in blue color and cells marked with green fluorescence were confirmed as positive cells.

In immunofluorescence analysis, anti-Eg-Fis1 and anti-Eg-PDCD6 rabbit IgGs were applied for detection of their respective native proteins in adult worms, cyst walls (from fertile and infertile cysts), and PSCs in *E. granulosus*. The results indicated that both Eg-Fis1 and Eg-PDCD6 were distributed in all tissues of *E.*, including the inner body and tegument of adult worms, the germinal layer (GL) of the cyst wall, and the parenchymal region and tegument of PSCs, but both were absent from the laminated layer (LL) of the cyst wall (Figure 4).

### 2.4. Expression of Eg-Fis1 and Eg-PDCD6 in Response to Oxidative Stress

According to previous studies, clear indications of apoptosis were apparent in PSCs following treatment with H_2_O_2_, and the degree of apoptosis increased gradually with increasing treatment time [19]. In our present study, PSCs were treated with 10 mM H_2_O_2_ for 8 h to induce apoptosis, and collected every 2 h. Quantitative PCR (qPCR) was then performed to assess Eg-Fis1 and Eg-PDCD6 mRNA expression levels in PSCs using β-actin (*actb*) as a reference gene.

**Figure 5:**
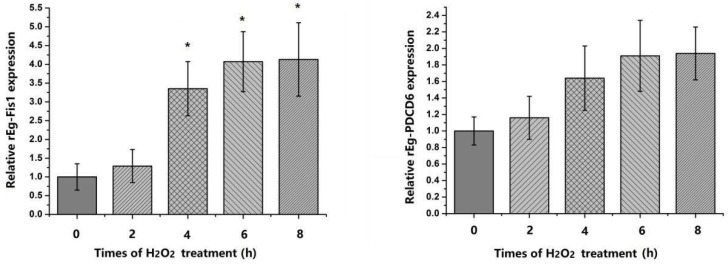
Expression profile analysis of Eg-Fis1 and Eg-PDCD6 in response to H_2_O_2_ treatment. Data are presented as the mean ± SD of triplicate experiments. Statistically significant differences between the 0 h group (control) and the other groups were determined using Student’s *t*-tests (* *p* < 0.05).

The results showed that Eg-Fis1 mRNA expression was upregulated at the beginning of H_2_O_2_ treatment, and levels continued to increase by up to 4-fold after an 8 h incubation. The mRNA levels were also gradually upregulated with increasing treatment time, but not significantly so (Figure 5).

### 2.5. Small Interfering RNA (siRNA)-Mediated Knockdown of Eg-Fis1 and Eg-PDCD6

Specific siRNAs were applied by soak transfection to silence target gene transcription, and the transfection efficiency was estimated to be ~60%, as determined by the control siRNA (siRNA-CON) and the 6-carboxyfluorescein (FAM) fluorescence label.

After two transfections, Eg-Fis1 and Eg-PDCD6 mRNA levels were determined by qPCR, as described above. The results showed that Fis-12, Fis-253, and Fis-364 decreased Eg-Fis1 expression by 59.60%, 36.77%, and 43.13%, respectively, while PDCD-153, PDCD-286, and PDCD-375 targeting of *Eg-PDCD6* decreased expression by 67.21%, 38.60%, and 46.12%, relative to siRNA-CON. Thus, Fis-253 and PDCD-286 were the most efficient siRNAs for knockdown of Eg-Fis1 and Eg-PDCD6 transcripts, respectively.

### 2.6. Measurement of Apoptotic Rate in PSCs Following RNAi Treatment

**Figure 6:**
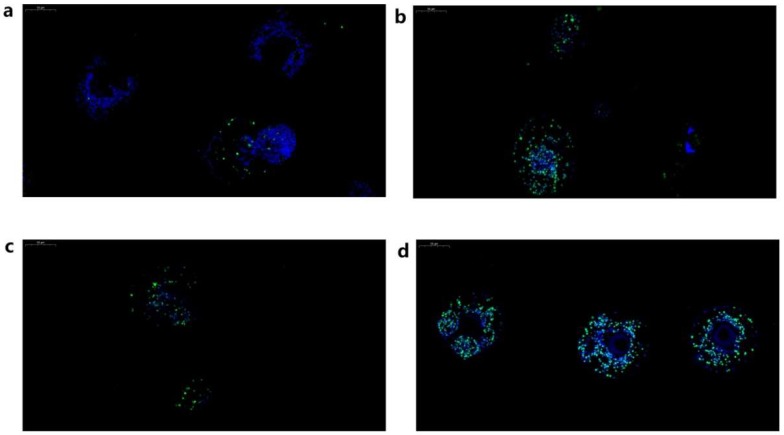
Terminal deoxynucleotidyl transferase dUTP nick end labelling (TUNEL) assay of *E. granulosus* PSCs following RNA interference (RNAi). (**a**) PSCs in the negative control group (NCG). (**b**) PSCs in the non-interference group (NIG); (**c**) PSCs in the Eg-Fis1 RNAi group (Fis-IG); (**d**) PSCs in the Eg-PDCD6 RNAi group (PDCD6-IG). TUNEL positive cells were stained in green, while negative cells were stained in blue.

To investigate whether Eg-Fis1 and/or Eg-PDCD6 are responsible for regulating the apoptosis response to oxidative stress, terminal deoxynucleotidyl transferase dUTP nick-end labelling (TUNEL) assays were performed (Table 1 and Figure 6). In the negative control group, the fluorescence signal from apoptotic cells in PSCs was minimal (13.95%, 42/301), but a significant increase in apoptotic rate was observed in all H_2_O_2_ treatment groups (*p* < 0.05). In the Eg-Fis1 RNAi group (Fis1-IG), the fluorescence signal from apoptotic cells was decreased after H_2_O_2_ treatment relative to the non-interference group (NIG); the apoptotic rate for the Fis1-IG group was 53.85% (182/338), compared with 63.87% (76/119) for the NIG. By contrast, the difference in fluorescence signal between the Eg-PDCD6 RNAi group (PDCD6-IG) and the NIG was not significant (*p* > 0.05); the apoptotic rate in the PDCD6-IG was 65.07% (285/438).

## 3. Discussion

Previous studies on the Fis1 gene have mainly concentrated on mammals, and reports on parasites are scarce [20]. PDCD6 is named after its role in the apoptosis of T cells, but the mechanism underpinning its role in the regulation of apoptosis is still unclear. Herein, we cloned, expressed, and characterised the Fis1 gene (*Eg-Fis1*) and PDCD6 gene (*Eg-PDCD6*) from *E. granulosus*, and explored their functions.

Our results showed that *Eg-Fis1* encodes a protein of 157 aa with a predicted MW of 16.93 kDa. Its N-terminal region was predicted to be exposed to the cytosol, and it likely adopts a novel tetratricopeptide repeat (TPR)-like helical bundle. This structure is known to stimulate mitochondrial fission and combine with other proteins [21]. In Fis1 mutant strains, mitochondrial fission was significantly suppressed, which demonstrates that Fis1 is required for the proper assembly, membrane distribution, and function of Dnm1-containing complexes during fission [22]. The transmembrane region of Fis1 is known to be very important for its function, since cytoplasmic localisation results in loss of the ability to promote mitochondrial fission when the transmembrane region is missing [8,9,10]. Multiple sequence alignment revealed that Fis1 genes were highly variable, and evolutionary analysis showed that Fis1 from *E. granulosus* shares higher homology with Fis1 from congeneric cestodes than with other species.

*Eg-PDCD6* encodes a polypeptide of 174 aa with a predicted MW of 20.34 kDa. Protein functional domain prediction revealed an EF-hand domain that likely facilitates binding to Ca^2+^ [23]. Indeed, PDCD6 (ALG-2) possesses two high-affinity Ca^2+^-binding sites, and binding alters the structure of the PDCD6 protein, exposing its hydrophobic region to allow it to interact with other proteins, facilitating a wider range of biological functions [24]. Moreover, *PDCD6* genes are highly conserved among species [13,25]. PDCD6 from humans is almost 100% identical to the mouse gene, and homologs are present in mammals and plants.

In our present study, localisation of Eg-Fis1 and Eg-PDCD6 in adult and larval stages of *E. granulosus* was confirmed by immunofluorescence, and both proteins were widely distributed in all tissues of *E. granulosus.* These findings suggest that Eg-Fis1 and Eg-PDCD6 might be involved in many important physiological functions in this organism.

In many cells, mitochondria are highly dynamic and regularly undergo fusion and fission [26], hence mitochondrial DNA and mitochondrial proteins can be continuously exchanged and distributed throughout the whole mitochondrial population [27,28,29]. Fis1 is the main mitochondrial fission factor in mammalian cells [10]. The wide distribution of Eg-Fis1 in *E. granulosus* is likely required to support normal life activities and metabolic needs.

Research has revealed novel functions of PDCD6 in addition to pro-apoptotic activity. Changes in protein conformation allow PDCD6 to interact with other proteins, and thereby induce various biological effects. Therefore, the widespread distribution of Eg-PDCD6 suggests that this protein might be involved in metabolic processes in *E. granulosus*.

In multicellular organisms, harmful stimuli can lead to excessive production of highly reactive molecules, such as reactive oxygen species (ROS) that cause oxidative damage [30], which can affect the structure and function of cells, causing tissue damage and pathological changes. Apoptosis is a fundamental biological phenomenon in multicellular organisms, and parasite load in hosts can be regulated by apoptosis to enhance host survival [31]. Previous studies demonstrated that oxidative DNA damage can exceed DNA repair mechanisms, triggering death of PSCs and apoptosis in the germinal layer, leading to infertility of hydatid cysts [19,32,33]. Thus, H_2_O_2_ was used as an apoptosis inducer to treat PSCs in our study, and expression profiles of Eg-Fis1 and Eg-PDCD6 following H_2_O_2_ exposure were analysed to further confirm whether these two proteins respond to oxidative stress. Expression of Eg-Fis1 was significantly upregulated with increasing duration of H_2_O_2_ treatment, and levels continued to increase up to 4-fold during an 8 h incubation. These results are similar to those of previous studies; Zhang and colleagues treated human lung cancer cells with UV irradiation to induce apoptosis, and Fis1 levels were slightly, but significantly, elevated after UV treatment [34]. This demonstrates that Fis1 is upregulated to mediate mitochondrial fission during UV irradiation-induced apoptosis. Furthermore, the mRNA expression level of Eg-PDCD6 was also upregulated, but the increase was not significant, suggesting that Eg-PDCD6 does not play a key role in the response to oxidative stress, although the functions of both genes requires further study.

To further confirm the functions of Eg-Fis1 and Eg-PDCD6 in PSCs during oxidative stress-induced apoptosis, RNAi experiments were performed, and the apoptotic rate in PSCs was determined. In the Eg-Fis1 RNAi group, the fluorescence signal from apoptotic cells was lower than the non-interference group (NIG), which indicates that lack of Eg-Fis1 could reduce apoptosis in *E. granulosus.* Fluorescence resonance energy transfer and coimmunoprecipitation analysis has already proven that Fis1 can interact with Drp1, and overexpression of human Fis1 causes mitochondrial fragmentation [8,9], while silencing of Fis1 leads to the extension and interconnection of mitochondria [10]. Since apoptosis induced by oxidative stress is one of the mechanisms causing infertility of hydatid cysts [19,32,33], further studies will be carried out to evaluate whether Eg-Fis1 is involved in regulating the production of infertile cysts.

By contrast, the difference in fluorescence signal between the Eg-PDCD6 RNAi group and NIG was not significant. To study the function of PDCD6 (ALG-2) under physiological conditions, Jang et al. generated PDCD6-deficient mice using a gene-targeting approach, and the results indicated that the general development and survival of mutant mice, as well as their immune system development and differentiation, was normal, consistent with our current results. Of interest, TCR-, Fas-, and dexamethasone-induced apoptosis of T-cells was not significantly impaired in the absence of PDCD6 [15]. We therefore speculate that Eg-PDCD6 is unlikely to be a key regulator of apoptosis.

## 4. Materials and Methods

### 4.1. Ethics Statement

All experimental procedures in the present study were reviewed and approved by the National Institute of Animal Health Animal Care and Use Committee of Sichuan Agricultural University (Ya’an, China, approval number: 2013-028, 25 December 2013). All animal experiments were performed by Dashuo Biological Technology Co., Ltd. (Chengdu, China) and animals were raised strictly in accordance with the Regulations for the Administration of Affairs Concerning Experimental Animals (approved by the State Council of the People’s Republic of China).

### 4.2. Animals and Parasites

*E. granulosus* (G1 genotype) cysts were collected from the livers of infected sheep at a local abattoir in Sichuan Province, China. Cysts containing PSCs in hydatid fluid were confirmed as fertile, while the rest were identified as infertile [35]. Cyst walls (including the GL and LL) from fertile and infertile cysts and PSCs were separated and treated as previously described [36]. Fresh PSCs at a concentration of 2000 mL^−1^ were cultured in complete RPMI 1640 medium (Hyclone, Logan, UT, USA), containing 100 μg·mL^−1^ streptomycin, 100 U·mL^−1^ penicillin G (Sigma, St. Louis, MO, USA), and 10% fetal calf serum (Hyclone), in an atmosphere containing 5% CO_2_ at 37 °C. A four-month-old dog was artificially infected with 30,000 PSCs, and adult worms were obtained from the small intestine after 35 days.

### 4.3. Bioinformatic Analysis

The cDNA sequences of Eg-Fis1 (EgrG_000225700) and Eg-PDCD6 (EgrG_000715700) were obtained from GeneDB (http://www.genedb.org/Homepage). Bioinformatic analyses included basic physicochemical properties, secondary structure, and functional prediction, as described previously [37]. Sequences were aligned, and phylogenetic trees were constructed using MEGA software (version 5.05) using the neighbour-joining (NJ) method.

### 4.4. Cloning and Expression of Recombinant rEg-Fis1 and rEg-PDCD6

Total parasite RNA was isolated from PSCs using TRIzol reagent (Tiangen, Beijing, China) following the manufacturer’s instructions, and cDNAs were synthesised using a Reverse Transcription System (Thermo Fisher, Waltham, MA, USA). The sequence encoding Eg-Fis1 was amplified by PCR with sense primer 5′-CGCGGATCCATGGAATTACTGGATTTGAATG-3′ and antisense primer 5′-CCGCTCGAGTTATTGTCCTTTGCCCTTCT-3′ containing *BamH*I and *Xho*I restriction enzyme sites, respectively. Eg-PDCD6 was amplified from cDNAs using sense primer 5′-CGCGGATCCATGAGCCAACCCTTTTCG-3′ containing a *BamH*I site, and antisense primer 5′-CCGCTCGAGTTATGTGAACACGGTAAAGACAC-3′ containing an *Xho*I site. PCR cycling involved an initial denaturation for 5 min at 94 °C followed by 35 cycles at 95 °C for 45 s, 60 °C for 30 s, and 72 °C for 45 s. Recombinant Eg-Fis1 and Eg-PDCD6 were cloned into pET-32a, expressed, and purified as described in our previous study [38]. Purified rEg-Fis1 and rEg-PDCD6 were separated by 15% SDS-PAGE, and the final protein concentration was determined using a BCA protein assay kit (Beyotime, Shanghai, China).

### 4.5. Preparation of Polyclonal Antibodies

Polyclonal antibodies against rEg-Fis1 and rEg-PDCD6 were produced from four 3-month-old female New Zealand white rabbits using an immunisation strategy described previously [38]. Rabbit sera were collected before and after immunisation and the antibody titre was determined. Afterwards, IgGs were isolated from sera using a protein G Sepharose column (Bio-Rad, Richmond, VA, USA), following the manufacturer’s instructions.

### 4.6. Immunoblotting and Immunolocalisation

Total PSC extracts and purified rEg-Fis1 and rEg-PDCD6 were separated by 15% SDS-PAGE and subsequently transferred to a nitrocellulose membrane (Millipore, Schwalbach, Germany) and blocked with 5% (*w*/*v*) skimmed milk, then incubated with *E. granulosus* positive/negative sheep sera, anti-rEg-Fis1/anti-rEg-PDCD6 rabbit sera, or pre-immunised rabbit sera (1:150 *v*/*v* dilutions) in 0.01 M phosphate-buffered saline (PBS). After four washes, membranes were incubated with horseradish peroxidase (HRP)-conjugated sheep anti-rabbit antibody, and signals were detected using diaminobenzidine (DAB) reagent (Tiangen, Beijing, China). 

Fertile and infertile cyst walls, PSCs, and adult worms were fixed for 36 h with 4% paraformaldehyde, embedded in paraffin, and sliced into sections. Pre-treatment of sections was implemented as described previously [38] followed by incubation with anti-Fis1/anti-rEg-PDCD6 rabbit IgG or native rabbit IgG (1:500 *v*/*v* dilutions in PBS) at 4 °C overnight. Sections were then incubated with fluorescein isothiocyanate (FITC)-conjugated sheep anti-rabbit IgG (1:200 *v*/*v* dilution in 0.1% Evan’s Blue) in the dark at 37 °C for 1 h, glycerine was added after washing four times with PBS, and images were collected using a fluorescence microscope (Nikon 55i, Tokyo, Japan).

### 4.7. Eg-Fis1 and Eg-PDCD6 Expression in PSCs Following H_2_O_2_ Treatment 

In 6-well microplates, PSCs were cultured for 2 days then treated with 10 mM H_2_O_2_ for 8 h to induce apoptosis. PSCs were collected every 2 h and placed immediately in liquid nitrogen until further study.

Quantitative real-time reverse-transcription PCR (qPCR) was carried out to evaluate Eg-Fis1 and Eg-PDCD6 transcript levels in PSCs in different states. RNA extraction and cDNA synthesis were conducted as described above. Primers for *Eg-Fis1* were 5′-TAACCTTCTCGCCGTCAATC-3′ and 5′-CACCGACAGCTAAACCAACA-3′, and primers for *Eg-PDCD6* were 5′-CTTATGATGTGCCGCTTTGA-3′ and 5′-CGAACCCTATCTGAATGTAGCC-3′. Primers for the internal reference β-tubulin (*actb*) housekeeping gene were 5′-ATGGTTGGTATGGGACAAAAGG-3′ and 5′-TTCGTCACAATACCGTGCTC-3′. Amplification was performed as described previously [38], all experiments were carried out in triplicate, and values were calculated using the 2^−ΔΔ*C*t^ method.

### 4.8. RNA Interference Assay

To further investigate the role of Eg-Fis1 and Eg-PDCD6 in PSCs in *E. granulosus*, expression levels were knocked down by RNAi. To target the cDNA sequences of Eg-Fis1 and Eg-PDCD6, three specific small interfering RNAs (siRNAs) were respectively designed and synthesised by GenePharma Co., Ltd. (Shanghai, China). We also synthesised a random siRNA with the FAM fluorescence label to determine the transfection efficiency (Table 2).

PSCs for which the survival rate was >95% were divided into seven groups. In each group, ~4000 PSCs were cultured in 3 mL of RPMI 1640 medium, and treated with 10 μL of transfection reagent (Engreen Biosystem, Beijing, China) plus the specific or random siRNA at a final concentration of 200 μM at days 0 and 2. Medium was replaced every day.

Eg-Fis1 and Eg-PDCD6 mRNA levels were determined by qPCR as described above. PSCs were collected for RNA isolation and cDNA synthesis at 24 h after the final addition of RNAi.

Afterwards, PSCs treated with the most efficient siRNA were selected and incubated with 10 mM H_2_O_2_ for 8 h in an atmosphere containing 5% CO_2_ at 37 °C (interference group, IG). PSCs treated with siRNA-CON were also incubated with H_2_O_2_ under the same conditions (non-interference group, NIG). After treatment, PSCs were collected and stored in liquid nitrogen for further study. A negative control group (NCG) cultured without any siRNA and H_2_O_2_ was also included.

### 4.9. Terminal Deoxynucleotidyl Transferase dUTP Nick End Labelling (TUNEL) Assay

After 8 h of exposure, PSCs were collected and prepared for TUNEL assays as described previously [39] and assays were performed using an In Situ Cell Death Detection Kit (Roche, Basel, Switzerland) following the manufacturer’s instructions. Samples were eventually stained with 1 μg·mL^−1^ of 4′,6-diamidino-2-phenylindole (DAPI) reagent (Sigma-Aldrich, St. Louis, MO, USA) at room temperature for 10 min, and cells marked with green fluorescence were confirmed as positive cells. The apoptotic rate of cells in PSCs was calculated as the percentage of positive cells (the number of positive cells/total cell number × 100) using Image-Pro Plus 6.0 (Media Cybernetics, Inc., Rockville, MD, USA).

### 4.10. Statistical Analysis

All data are presented as mean ± standard deviation (SD). For comparison between groups, Student’s unpaired *t*-tests and one-way analysis of variance (ANOVA) were performed using SPSS 13.0, and *p*-values < 0.05 were considered statistically significant.

## 5. Conclusions

In this study, Eg-Fis1 and Eg-PDCD6 were cloned, expressed, and characterised. Both Eg-Fis1 and Eg-Fis1 were ubiquitously distributed in adult worms, PSCs, and the GL of the cyst wall. Expression of both Eg-Fis1 and Eg-PDCD6 was upregulated after triggering of apoptosis following H_2_O_2_ treatment, but the increase in Eg-PDCD6 expression was not significant. In addition, the apoptotic rate was significantly decreased after RNAi knockdown of Eg-Fis1, but there was no difference between the Eg-PDCD6 RNAi group and the non-interference group. In conclusion, Eg-Fis1 promotes apoptosis induced by oxidative stress in *E. granulosus*, and thereby stimulates the production of infertile cysts, whereas Eg-PDCD6 does not appear to be a key regulator of apoptosis.

## Figures and Tables

**Table 1:** Apoptosis rate of PSCs determined by TUNEL assay.

Group Name	Number of Apoptotic Cells	Total Cells	Apoptosis Rate (%)
NCG	42	301	13.95%
NIG	76	119	63.87%
Fis1-IG	182	338	53.85%
PDCD6-IG	285	438	65.07%

NCG, negative control group; NIG, non-interference group; Fis1-IG, Eg-Fis1 RNAi group; PDCD6-IG, Eg-PDCD6 RNAi group.

**Table 2:** Sequences of small interfering RNAs.

siRNA Name	Sequences	
	Forward (5′-3′)	Reverse (5′-3′)
FIS-12	GGAUUUGAAUGAGCCGGUUTT	AACCGGCUCAUUCAAAUCCTT
FIS-253	GCAAUCGAAUGUUGCGAUATT	UAUCGCAACAUUCGAUUGCTT
FIS-364	GCUGUUGGUUUAGCUGUCGTT	CGACAGCUAAACCAACAGCTT
PDCD-153	CGACCUCUUUAGUCCUAAUTT	AUUAGGACUAAAGAGGUCGTT
PDCD-286	GAGCUACAGUCCGCUAUAUTT	AUAUAGCGGACUGUAGCUCTT
PDCD-375	GGGCGUUGUUUACUUCGAUTT	AUCGAAGUAAACAACGCCCTT
siRNA-CON	UUCUCCGAACGUGUCACGUTT	ACGUGACACGUUCGGAGAATT
